# Genome-Wide Analysis and Characterization of Eggplant F-Box Gene Superfamily: Gene Evolution and Expression Analysis under Stress

**DOI:** 10.3390/ijms232416049

**Published:** 2022-12-16

**Authors:** Yixi Wang, Chuhao Li, Shuangshuang Yan, Bingwei Yu, Yuwei Gan, Renjian Liu, Zhengkun Qiu, Bihao Cao

**Affiliations:** 1 Key Laboratory of Biology and Genetic Improvement of Horticultural Crops, Ministry of Agriculture and Rural Affairs, College of Horticulture, South China Agricultural University, Guangzhou 510642, China; 2 Guangdong Vegetable Engineering and Technology Research Center, Guangdong Provincial Key Laboratory of Postharvest Science of Fruits and Vegetables, South China Agricultural University, Guangzhou 510642, China; 3 Guangdong Province Key Laboratory of Microbial Signals and Disease Control, Integrative Microbiology Research Centre, South China Agricultural University, Guangzhou 510642, China

**Keywords:** eggplant, F-box superfamily, genome-wide analysis, stress, functional identification

## Abstract

F-box genes play an important role in plant growth and resistance to abiotic and biotic stresses. To date, systematic analysis of F-box genes and functional annotation in eggplant (*Solanum melongena*) is still limited. Here, we identified 389 F-box candidate genes in eggplant. The domain study of F-box candidate genes showed that the F-box domain is conserved, whereas the C-terminal domain is diverse. There are 376 *SmFBX* candidate genes distributed on 12 chromosomes. A collinearity analysis within the eggplant genome suggested that tandem duplication is the dominant form of F-box gene replication in eggplant. The collinearity analysis between eggplant and the three other species (*Arabidopsis thaliana*, rice and tomato) provides insight into the evolutionary characteristics of F-box candidate genes. In addition, we analyzed the expression of *SmFBX* candidate genes in different tissues under high temperature and bacterial wilt stress. The results identified several F-box candidate genes that potentially participate in eggplant heat tolerance and bacterial wilt resistance. Moreover, the yeast two-hybrid assay showed that several representative F-box candidate proteins interacted with representative Skp1 proteins. Overexpression of *SmFBX131* and *SmFBX230* in tobacco increased resistance to bacterial wilt. Overall, these results provide critical insights into the functional analysis of the F-box gene superfamily in eggplant and provide potentially valuable targets for heat and bacterial resistance.

## 1. Introduction

The ubiquitin/26S proteasome system (UPS) can degrade about 80% to 90% of the proteins in plant cells to maintain plant stability and development [1]. The UPS primarily includes ubiquitin, ubiquitin-activating enzyme E1, ubiquitin-conjugating enzyme E2, ubiquitin E3 ligase, and the 26S proteasome [2]. Among them, E3 ligase is the core component of UPS, which can specifically recognize the target proteins [3]. SCF-type cullin RING ubiquitin E3 ligases (CRL^SCF^), the most abundant multi-subunit E3 ligases, are primarily composed of the backbone cullin 1 protein (CUL1), E2-linked RING protein RBX1, adaptor Skp1, and substrate receptor F-box [4,5]. The F-box domain was first discovered at the N-terminal of cyclin F [6]. The typical F-box proteins usually contain an F-box domain (40–50 bp) at the N-terminus that binds to the Skp1 subunit and a diverse C-terminal domain that can bind to target proteins [7].

F-box genes are numerous and diverse. The F-box gene superfamily has been reported in many plant species, including *Arabidopsis thaliana* [8], rice (*Oryza sativa*) [9], soybean (*Glycine max*) [10], maize (*Zea mays*) [11], alfalfa (*Medicago sativa*) [12], chickpea (*Cicer arietinum*) [13], apples (*Malus pumila*) [14], pear (*Pyrus* spp.) [15], upland cotton (*Gossypium hirsutum*) [16], barley (*Hordeum vulgare*) [17], and tomato (*Solanum lycopersicum*) [18], which contain 568, 687, 509, 359, 972, 285, 517, 226, 592, 126, and 139 F-box genes, respectively. There are usually more F-box genes in plants than in animals. For example, humans and mice only have 68 and 74 F-box genes, respectively [19]. Most F-box classifications are based on the types of protein C-terminal domains, which has resulted in a large number of subfamilies. In the previous classification, plant F-box gene families, including *A. thaliana* [8], rice [9], soybean [10], corn [11], alfalfa [12], chickpeas [13], apples [14], pear [15], upland cotton [16], and barley [17], were classified into 19, 10, 9, 15, 15, 10, 12, 12, 17, and 9 subfamilies, respectively. However, contrary to the previous classification, tomato was recently divided into only five subfamilies based on phylogenetic analysis [18].

F-box genes are crucial in plant resistance to abiotic and biotic stresses. For example, Zhou et al. [20] showed that the wheat (*Triticum aestivum*) F-box protein TaFBA1, which contains the AMN1 domain at the C-terminus, can improve the ability of plants to resist oxidative stress and positively regulates resistance to drought [21]. In addition, TaFBA1 is associated with heat and salt tolerance in wheat [22,23]. In *A. thaliana* [24] and apple [25], the F-box protein MAX2 can positively modulate tolerance to drought and salt stress by regulating endogenous abscisic acid (ABA) signaling. With regard to disease resistance, both MAX2 and the F-box-Nictaba proteins can positively regulate the resistance to *Pseudomonas syringae* in *A. thaliana* [26,27]. The F-box protein GhACIF1 increases resistance to *Verticillium dahliae* through hypersensitive and adaptive immune responses in upland cotton [28]. There are also reports of negative regulation by F-box genes. For instance, the F-box protein CPR30 is a negative regulator of the R protein SNC1 and negatively modulates resistance to plant disease by regulating salicylic acid (SA) signaling [29,30].

Eggplant (*Solanum melongena*) is an important solanaceous crop. Data from the Food and Agriculture Organization (FAO, https://www.fao.org, accessed on 1 September 2022) indicated that the global planting area of eggplant reached 1.846 million hectares in 2020, with a total output of approximately 56.302 million tons. However, eggplant production is hampered by abiotic and biotic stresses, particularly high temperatures and bacterial wilt (BW) [31,32]. BW is a global bacterial soilborne disease caused by *Ralstonia solanacearum*, which has a wide host range [33]. A previous study revealed that severe infection by BW can reduce the yield of eggplants by 50% to 60% [34]. It is worth noting that *R. solanacearum* is ranked second on the list of the top 10 most scientifically and economically costly plant pathogens [35]. However, there are far more *R. solanacearum* resistant and tolerant varieties in eggplant than in other solanaceous crops. The wild relatives of eggplant are essential sources for transferring tolerance to abiotic and biotic stresses [36]. One study reported that most wild species resist almost all known eggplant pests and diseases [37].

Although the F-box gene superfamily has been reported in many species, systematic analysis of the eggplant F-box gene superfamily is lacking. The completion of eggplant whole-genome sequencing in recent years has enabled analysis of the eggplant F-box gene superfamily [38]. This study identified 389 F-box candidate genes from the eggplant genome and analyzed their structure and evolution. We analyzed the expression pattern of eggplant F-box candidate genes and screened out the genes associated with resistance to heat stress and BW. A yeast two-hybrid assay was also applied to explore the interaction between representative SmFBX proteins and Skp1 proteins. In addition, we evaluated the functions of SmFBX131 and SmFBX230 proteins through transient overexpression experiments in *Nicotiana benthamiana*. Overall, this analysis of the eggplant F-box gene superfamily lays a foundation to better understand the regulatory network of eggplant disease and stress resistance.

## 2. Results

### 2.1. Identification of Eggplant F-Box Candidate Genes

A combination of HMM and PSI-BLAST was used to identify F-box candidate genes in eggplant. Many genes that were annotated as F-box genes in the eggplant genome could not be detected by HMM only, which indicated that the method was not sensitive enough. Thus, we used PSI-BLAST to align each amino acid sequence to a database composed of F-box proteins of four species identified by HMM. Results identified 389 F-box candidate proteins (26% more than when only using HMM) in eggplant (Supplementary Table S1). In addition, we identified 750, 728, and 399 F-box candidate proteins in *A. thaliana*, rice, and tomato, respectively (Supplementary Files S1–S4). No more F-box candidate genes were identified after 10 rounds of PSI-BLAST iterations (Supplementary Figure S1). Importantly, compared to previous studies, we identified 111 new F-box candidate proteins in *Arabidopsis* that were phylogenetically related to the previously identified ones, indicating the robustness of our method (Supplementary Figure S2).

### 2.2. Conserved Site Analysis of the F-Box Domain

To investigate the conserved sites of the eggplant F-box domain, we aligned the F-box domain sequence of the 389 F-box candidate proteins. The two most abundant non-gap amino acids at each position were defined as conserved. A total of 19 conserved residues were identified, with the highly conserved residues being Ile-36 (position) (293, 75.32%), Trp-80 (270, 69.41%), Leu-42 (267, 68.64%), L-16 (265, 68.12%), Pro-17 (258, 66.32%), Val-76, (252, 64.78%), Leu-37(232, 59.64%), and Lys-78, (224, 57.58%). In addition, the F-box conserved residues in *A. thaliana*, rice, and tomato were aligned, and the number of these conserved residues was 17, 18, and 19, respectively. Results revealed 60.83% F-box conserved residues between the four species (*A. thaliana*, rice, tomato, and eggplant) (Supplementary Figure S3A–D, Supplementary File S5). A total of 20 F-box candidate protein sequences from the four species were randomly selected and aligned with the human F-box protein Skp2. We found that most of the residues were conserved. The binding site in Skp2 was mainly consistent with the conserved residues, indicating that the conserved residues may be the binding site of F-box and Skp1 protein (Supplementary Figure S3E).

### 2.3. Analysis of the C-Terminal Domain of F-Box Candidate Proteins

**Figure 1:**
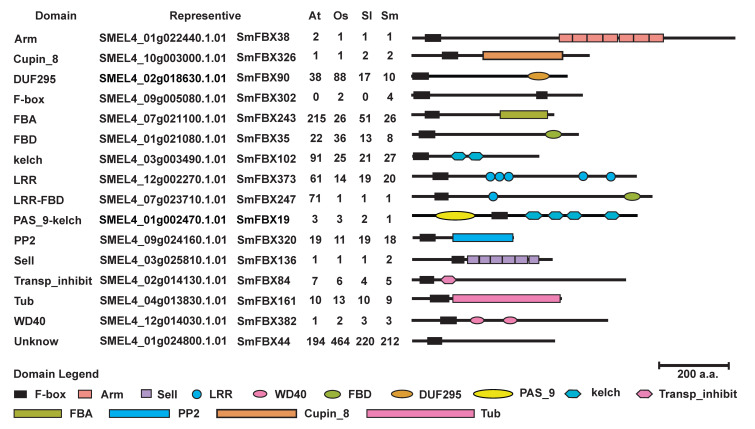
Schematic representation of representative F-box domains. A total of 16 representative F-box proteins were screened in *Arabidopsis thaliana*, rice (*Oryza sativa*), tomato (*Solanum lycopersicon*), and eggplant (*S. melongena*). The domain represents the type of C-terminal domain of the F-box candidate protein and counts the predicted number in the four species.

Most F-box proteins contain a functional domain at the C-terminus that recognizes proteins. To explore the regularity of F-box C-terminal domains in eggplant, the numbers and types of F-box C-terminal domains in eggplant, *A. thaliana*, rice, and tomato were counted. Results revealed that some C-terminal domain types were abundant in the F-box candidate proteins of the four species, including LRR, Kelch, FBD, and FBA. On the other hand, some C-terminal domain types, such as Arm, sel1, and Cupin_8, were less abundant. Notably, many FBXUs (F-box candidate genes with unknown C-terminal domain) were present in all four species (Figure 1, Supplementary Table S2). The ubiquitous F-box C-terminal domain in the four species could be involved in the basic physiological and biochemical regulation of plants. Different species also have special C-terminal domains, such as DLH, FMO-like, GRAS, FBA_RVT, and FBD_Tcp11 in eggplant; LRR_PPR_RVT in *A. thaliana*, HORMA in rice, and TPR_8_Sel1 domain in tomato (Supplementary Table S2). These domains may be associated with the specificity of different species.

### 2.4. Chromosomal Location of the Eggplant F-Box Candidate Genes

A chromosomal map was constructed to explore the location of eggplant F-box candidate genes on the chromosomes. It was found that all the F-box candidate genes were distributed on 12 chromosomes. However, 13 F-box genes (SmFBX1-13) were not mapped on any successfully spliced eggplant chromosome (chromosome 0). In addition, most of the F-box candidate genes were located at the ends of the chromosome arms (Supplementary Figure S4).

### 2.5. Duplication and Collinearity Analysis of the F-Box Candidate Genes

To study the evolutionary mechanism of eggplant F-box candidate genes, genes with 90% homology were defined as duplicate genes. We identified 15.68% (61/389) F-box candidate repeat genes in eggplant that were divided into 26 groups (Supplementary Table S3). In addition, the ratios of non-synonymous (Ka) versus synonymous (Ks) mutations (Ka/Ks) were analyzed. Results showed that the Ka/Ks ratios of 46 F-box candidate duplicate gene pairs ranged from 0 to 3.32, with an average of 0.76. In addition, the Ka/Ks values of eight repeated genes were >1, whereas those of the 30 repeat genes were <1 (Supplementary Table S4). These results suggested that the F-box candidate repeat genes were conserved. Most gene pairs had undergone purifying selection pressure, and a few repeat genes had undergone positive selection. We also explored the expression patterns of the F-box candidate duplicate genes in eggplant. It was found that most F-box candidate duplicate genes that belonged to the same group had a similar expression pattern. In contrast, some groups, such as *SmFBX382* and *SmFBX217*, and *SmFBX331* and *SmFBX333*, were differentially expressed (Supplementary Table S3), suggesting that the duplicate genes could confer new functions.

A collinearity analysis was performed to explore the duplication model of eggplant F-box candidate duplication genes (Supplementary Figure S5A; Supplementary Table S3). Results indicated at least seven pairs of candidate duplicated genes on different chromosomes, and at least 14 pairs of candidate duplicated genes on the same chromosome (Supplementary Figure S5A). According to Holub [39], repetitive genes that occur within 200 kb of the same chromosome are defined as tandem duplications. In this study, we found that 60% of the eggplant F-box candidate duplicated genes were tandem duplications (Supplementary Figure S6), suggesting that tandem repeats were the source of the most recently duplicated genes. In addition, the remaining 40% of duplication events include transposon-mediated duplication, segmental duplication, and retroduplication.

To further investigate the evolutionary mechanisms of the eggplant F-box candidate genes, three collinear analyses were conducted between eggplant and three representative species, including *A. thaliana*, rice, and tomato (Supplementary Figure S5B–D). There were 215 orthologous pairs between eggplant and tomato, 81 between eggplant and *A. thaliana*, and 47 between eggplant and rice. Compared with tomato, the locations of F-box candidate genes on eggplant chromosomes 1, 6, and 7 were almost unchanged. However, there were also cases where the positions of homologous F-box candidate genes had been exchanged at both ends of the eggplant and tomato chromosomes or directly transferred to the other chromosome of eggplant or tomato, such as the F-box homologous genes on eggplant chromosomes 4, 5, 8, 9, 10, and 11 (Supplementary Figure S5D). Collectively, these results suggest that chromosome rearrangement was the primary reason for the change in the chromosomal position of the F-box candidate genes in eggplant and tomato.

### 2.6. Analyzing the Expression of F-Box Candidate Genes in Eggplant Tissues

**Figure 2:**
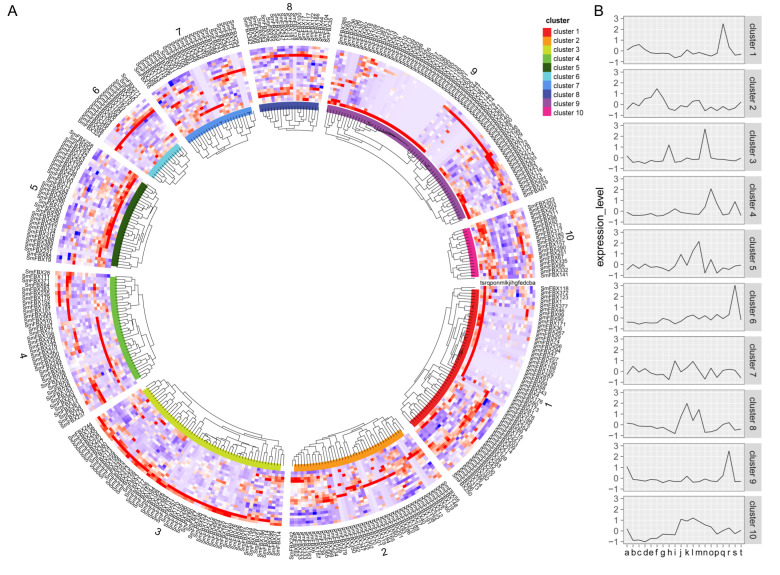
Transcriptome analysis of the F-box candidate gene superfamily in each tissue site of eggplant. (**A**) The expression in 20 eggplant tissues was measured and is represented by 20 letters (a–t) in cluster 1, which represents the roots (a), roots inoculated with *Verticillium dahliae* at 6 hpi (hours post-inoculation) (b), flowers (c), pistils (d), radicles (e), leaves (f), cotyledons (g), fruit stage 1 (h), fruit 1 cm (i), fruit 6 cm (j), fruit flesh stage 2 (k), stems (l), fruits skin stage 2 (m), fruit calyx stage 2 (n), senescent leaves (o), fruit peduncle (p), fruit skin stage 3 (q), fruit flesh stage 3 (r), buds 0.7 cm (s), and opened buds (t). Differently colored blocks represent different clusters. (**B**) The F-box was divided into 10 clusters, and the model for expression was obtained after counting the expression patterns of the F-box candidate genes in each cluster.

The gene expression patterns can provide insight into the functions of genes. To investigate the expression of F-box candidate genes in eggplant tissues, we downloaded transcriptome data obtained from 20 eggplant tissues from the National Center for Biotechnology Information (NCBI) and analyzed it. Among them, 229 F-box candidate genes were ubiquitously expressed in 20 tissues and developmental stages, 118 F-box candidate genes were specifically expressed in tissues, and 64 F-box candidate genes were expressed at low levels. The F-box candidate genes were divided into 10 clusters based on their expression patterns (Figure 2, Supplementary Tables S5 and S6). We found that the F-box candidate genes in cluster 3 were highly expressed in roots inoculated with *V. dahliae* for 6 hours (h). The top three domain types in this cluster were FBXU (number of members: 19), Kelch (10), and LRR (6). The F-box candidate genes in cluster 1 were highly expressed in buds, and the F-box candidate genes in cluster 9 were highly expressed in open buds and flowers. Notably, the top three domain types in clusters 1 and 9 were the same, including FBXU (cluster: number of members; cluster 1: 27, and cluster 9: 38), FBA (cluster 1: 5 and cluster 9: 9), and DUF295 (cluster 1: 4 and cluster 9: 4). The F-box candidate genes in clusters 4 and 6 were highly expressed in mature leaves, with those in cluster 4 being more highly expressed in senescent leaves. FBA, PP2, and FBD were the primary components in clusters 4 and 6. In addition, the expression of F-box candidate genes in clusters 5 and 10 increased or decreased at all stages and parts of fruit growth and development (Figure 2, Supplementary Tables S5–S7). These results suggest that F-box candidate genes that are specifically and highly expressed in different tissues may play a tissue-specific function.

### 2.7. Expression Analysis of the F-Box Candidate Genes under Abiotic Stress

**Figure 3:**
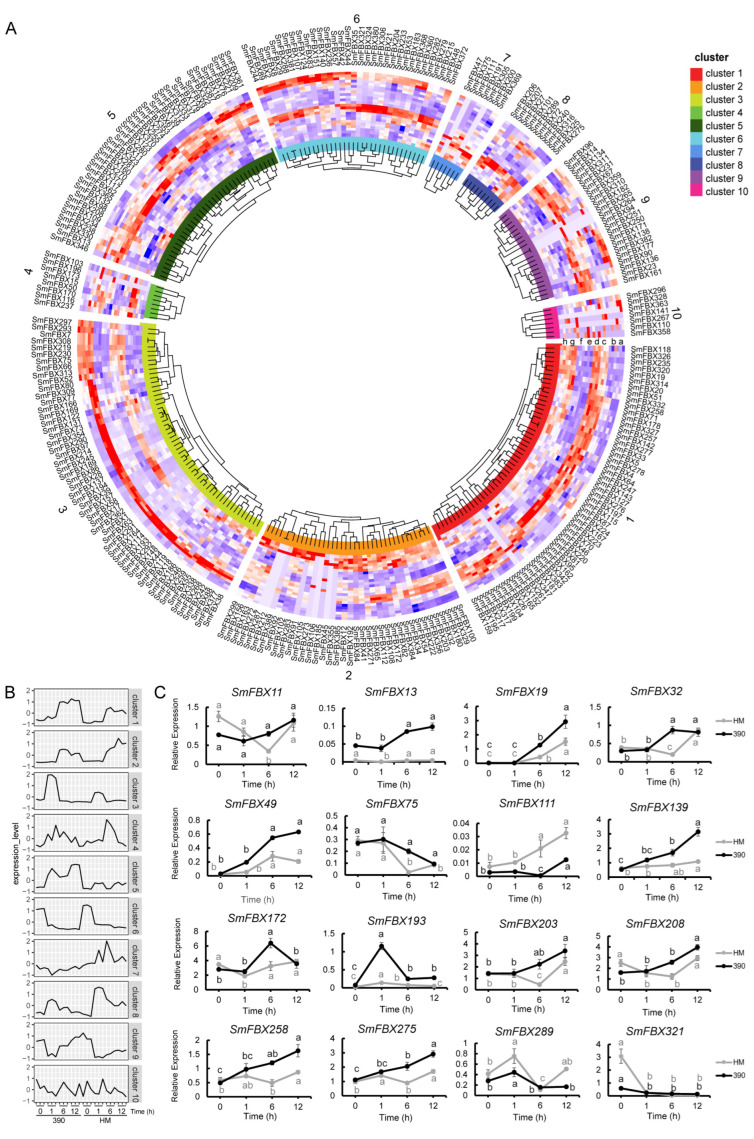
Transcriptome analysis and qRT-PCR verification results of the F-box candidate gene superfamily in eggplant heat-tolerant (390) and heat-sensitive (HM) varieties after high-temperature treatment. (**A**) F-box transcriptome heatmap analysis of eggplant heat-tolerant cultivar (390) and heat-sensitive cultivar (HM) after high-temperature treatment at 42 °C. The different treatments are represented by the eight letters (a–h) in cluster 1, with a-d indicating high-temperature treatment in 390 0, (a), 1 (b), 6 (c), 12 h (d); and e–h indicating high-temperature treatment in HM, 0 (e), 1 (f), 6 (g), 12 h (h). Different clusters are marked with different colors, and the inner circle part indicates phylogenetic analysis of the the F-box genes. There were three biological replicates for each treatment. (**B**) The F-box candidate genes were divided into 10 clusters, and the expression model was obtained after counting the expression patterns of the F-box genes in each cluster. (**C**) qRT-PCR validation of the screened F-box candidate genes. Both 390 and HM varieties were subjected to high temperature (42 °C) treatment, and samples were collected after 0, 1, 6, and 12 h. The calculation method was 2^−∆∆ct^ and the significant differences were analyzed using the least significant difference (LSD) for multiple comparisons, marked with letters. qRT-PCR, real-time quantitative reverse transcription PCR.

To explore the function of eggplant F-box candidate genes under abiotic stress, the expression patterns of the F-box candidate genes under high-temperature treatment (HTT) were analyzed from previous transcriptome data (Figure 3A,B, Supplementary Table S8). The F-box candidate genes in clusters 1 and 2 were highly induced after 6 h and 12 h of high-temperature treatment in the heat-resistant (390) and heat-sensitive (HM) varieties, respectively. The F-box candidate genes in cluster 3 first increased after 1 h of HTT and then decreased after 6 h and 12 h. In addition, FBXU (cluster: number of members; cluster 1: 23, cluster 2: 26, and cluster 3: 22), PP2 (cluster 1: 8, cluster 2: 1, and cluster 3: 7), and FBA (cluster 1: 4, cluster 2: 2, and cluster 3: 5) were the majority in clusters 1, 2, and 3. It was also found that the F-box candidate genes in cluster 6 were highly expressed before HTT but poorly expressed after HTT. Moreover, the F-box candidate genes in clusters 5 and 9 were highly expressed in 390 and poorly expressed in HM. FBXU had the highest expression, whereas Kelch (clusters 5: 4 and 9: 6), LRR (clusters 5: 6 and 9: 2), and FBA (cluster 5: 5) had the second highest expression (Figure 3A,B, Supplementary Tables S8–S10). These clusters of F-box candidate genes may be involved in plant regulation or response to temperature stress.

A total of 16 F-box candidate genes were selected for real-time quantitative reverse transcription PCR (qRT-PCR) verification. The results showed that the expression patterns of 16 F-box candidate genes were consistent with those from the transcriptome data. The expression levels of *SmFBX13*, *SmFBX19*, *SmFBX49*, *SmFBX139*, *SmFBX203*, *SmFBX208*, *SmFBX258*, and *SmFBX275* were increased by HTT in 390, whereas those of *SmFBX75* and *SmFBX321* were decreased by HTT in both varieties. We also found that *SmFBX11*, *SmFBX32*, *SmFBX172*, and *SmFBX193* were differentially expressed at different time points after HTT in the two cultivars. The expression patterns of *SmFBX111* and *SmFBX289* appeared to show a higher response in HM compared with 390 (Figure 3C). Except for *SmFBX97*, the other 15 genes were broadly expressed in tissues and developmental stages of eggplant (Supplementary Table S5). These results suggest that *SmFBX11*, *SmFBX13*, *SmFBX49*, *SmFBX172*, and *SmFBX193* positively respond to heat stress, whereas *SmFBX75*, *SmFBX111*, and *SmFBX289* negatively respond to heat stress in eggplant.

### 2.8. Analyzing the Expression of F-Box Candidate Genes under Biotic Stress

**Figure 4:**
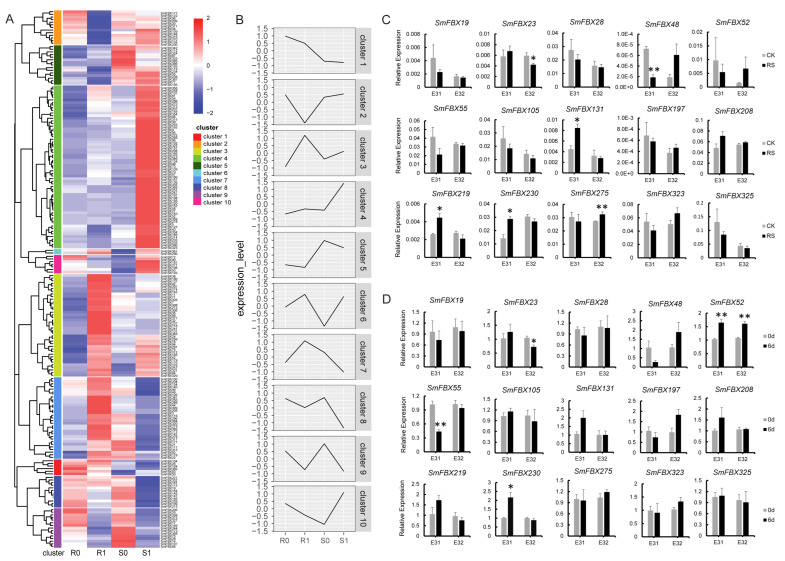
Transcriptome analysis of the F-box candidate gene superfamily in bacterial wilt resistant (E31, R) and susceptible (E32, S) eggplant varieties treated with *Ralstonia solanacearum,* followed by qRT-PCR verification. (**A**) F-box transcriptome heatmap analysis of E31 and E32 treated with *R. solanacearum*. R0 and S0 represent the disease-resistant and -susceptible eggplant varieties not inoculated with *R. solanacearum,* respectively. R1 and S1 represent the resistant and susceptible varieties after inoculation with *R. solanacearum*. Different clusters are marked with varying colors, and the left part of the figure indicates the phylogenetic analysis of the F-box candidate genes. (**B**) The F-box candidate genes were divided into 10 clusters, and the model of expression was obtained by counting the expression patterns of the genes in each cluster. (**C**) qRT-PCR validation of the screened F-box candidate genes. The expression analysis of the screened *SmFBX* candidate genes in each treated plant on the 6th day after *R. solanacearum* infection of E31 and E32 varieties. Samples were obtained, the RNA was extracted, and qRT-PCR was performed to verify the expression of the F-box candidate genes. CK, uninfected plants. RS, plants infected with *R. solanacearum*. (**D**) qRT-PCR validation of the screened F-box candidate genes after infection by *R. solanacearum*. The bacterial wilt-resistant variety E31 and the susceptible variety E32 were sampled after 0 and 6 days of infection by *R. solanacearum.* The RNA was extracted and qRT-PCR was performed to verify the expression of F-box candidate genes. The method of calculation was 2^−∆∆ct^. qRT-PCR, real-time quantitative reverse transcription PCR. * *p* < 0.05. ** *p* < 0.01. Student’s *t*-test.

To explore the function of eggplant F-box candidate genes under biotic stress, we analyzed the expression patterns of the F-box candidate genes in eggplant after inoculation with *Ralstonia solanacearum* for seven days (Supplementary Tables S11 and S12). Results showed that the expression of the F-box candidate genes in clusters 2, 4, 7, and 10 changed significantly after *R. solanacearum* inoculation. The expression of F-box candidate genes in cluster 7 and part of cluster 3 increased in the eggplant BW-resistant variety E31 (R). The F-box candidate genes in cluster 10 (except for *SmFBX53*) were highly expressed in the eggplant BW-susceptible variety E32 (S) and poorly expressed in the BW-resistant variety E31. The F-box candidate genes in cluster 4 were highly expressed in E32, whereas the genes in cluster 2 were poorly expressed in E31 (Figure 4A,B). Except for the FBXU (number of members: 16), the LRR (4) domain comprised the largest proportion in cluster 7. The F-box candidate genes that lacked an LRR domain (excluding *SmFBX53*) were in clusters 2 and 10. Moreover, the LRR domain was the most common domain type in cluster 4, followed by the FBXU domain (Supplementary Table S13). These results suggest that the F-box candidate genes in clusters 3 and 7 positively respond to BW, whereas the F-box candidate genes in clusters 2, 4, and 10 negatively respond to BW.

A total of 15 F-box candidate genes were selected for expression analysis. The expression levels of *SmFBX48* and *SmFBX275* decreased in the BW-resistant line E31 and increased in the BW-susceptible line E32. The expressions of *SmFBX52*, *SmFBX197*, and *SmFBX208* remained unchanged in E31 but increased in E32. The expression levels of *SmFBX23*, *SmFBX105*, *SmFBX219*, and *SmFBX323* remained unchanged or increased in E31 but decreased in E32. The expression level of *SmFBX131* increased in E31 but remained unchanged in E32. The expressions of *SmFBX55* and *SmFBX230* decreased in E31 but remained unchanged in E32. In addition, the expression levels of *SmFBX19*, *SmFBX28*, and *SmFBX325* were the same in E31 and E32 (Figure 4A). It was evident that all genes, except for *SmFBX197,* were ubiquitously expressed in the tissue-specific transcriptome (Supplementary Table S4).

After six days of *R. solanacearum* inoculation, the expression level of *SmFBX48* was significantly decreased in E31, whereas *SmFBX219*, *SmFBX131*, and *SmFBX230* were upregulated in E31. The expression of *SmFBX275* increased, whereas *SmFBX23* expression decreased in E32 (Figure 4C). We also compared the expression levels of the F-box candidate genes in plants at 0 and 6 days after *R. solanacearum* inoculation. The results showed that the expression level of *SmFBX55* decreased in E31 but remained unchanged in E32. The expression of *SmFBX230* increased significantly in E31, whereas the expression of *SmFBX23* decreased in E32 but remained unchanged in E31 (Figure 4D). These analyses suggest that *SmFBX219*, *SmFBX131*, *SmFBX23*, and *SmFBX230* positively respond to BW, whereas *SmFBX48* and *SmFBX275* negatively respond to BW.

### 2.9. Some Eggplant F-Box Candidate Proteins Interact with Skp1 Proteins

**Figure 5:**
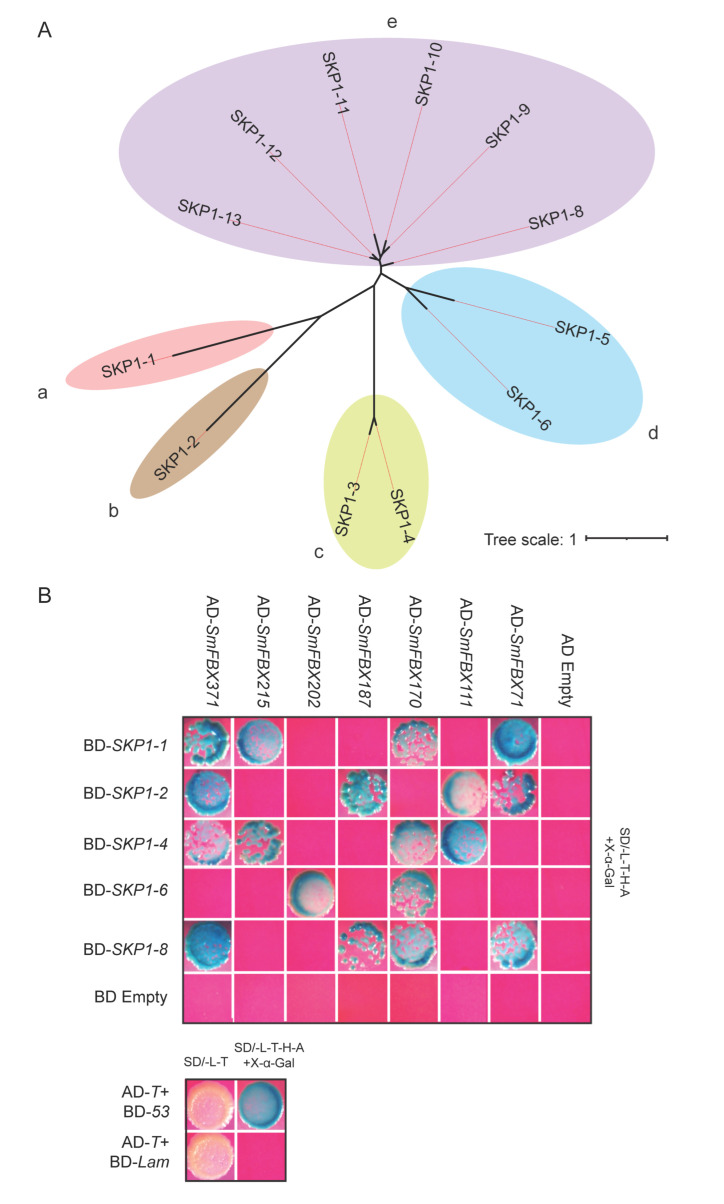
The representative Skp1s interacted with the F-box candidate proteins screened in the Y2H system. (**A**) The Skp1-like proteins in eggplant were screened, and an unrooted phylogenetic tree was constructed. Notably, a–e represent different subfamilies of Skp1-like proteins. Each subfamily is represented by a different color block. (**B**) Selected representative Skp1s interacted with the representative F-box candidate proteins. The F-box genes were constructed in the pGADT7 (AD) vector, and the Skp1-like genes were constructed in the pGBKT7 (BD) vector. After co-transforming Y2H Gold yeast competent cells, the yeast plaques could grow on the four-deficient plates, and the plaques could be stained blue by X-α-Gal. This suggests that the two proteins can interact with each other. The growth of yeast cells after AD-T co-transfection with BD-*53* or BD-*Lam* was the positive and negative control, respectively. SD/-L-T and SD/-L-T-H-A, SD media that lack two and four amino acids, respectively. Y2H, yeast hybrid assay.

Previous studies have shown that the F-box genes interact with Skp1 to form the SCF protein complex [6]. A total of 13 SKP1-like proteins were identified in eggplant and were named SKP1-1 to SKP1-13 (Supplementary Table S14, Supplementary File S6). The proteins were divided into five subfamilies (a–e) according to the phylogenetic analysis (Figure 5A). In addition, the 389 candidate *SmFBX* genes were divided into seven subfamilies (A–G) based on the phylogenetic analysis (Supplementary Figure S7). Five representative Skp1-like proteins (a subfamily: SKP1-1; b: SKP1-2; c: SKP1-4; d: SKP1-6; and e: SKP1-8) and seven representative SmFBX candidate proteins (A subfamily: SmFBX71; B: SmFBX317; C: SmFBX187; D: SmFBX202; E: SmFBX215; F: SmFBX170; and G: SmFBX111) were selected to perform a yeast two-hybrid (Y2H) assay. The fusion expression vector (pGADT7 and pGBKT7) that contained the two genes was transferred into yeast cells. The transformed yeast cells could form plaques on quadruple dropout medium (SD/-Leu-His-Trp-Ade) and turned blue following the addition of X-α-Gal, indicating that the two proteins could interact with each other. Herein, no activity was detected when either the BD-*SKP1* or AD-*SmFBX* fusion protein was expressed alone, and seven F-box candidate proteins could interact with at least one of the five Skp1-like proteins (Figure 5B). In addition, we found that varying types of Skp1 specifically interacted with different F-box candidate proteins. For example, SKP1-2 interacted with SmFBX71, SmFBX111, SmFBX187, and SmFBX371; and SKP1-6 interacted with SmFBX170 and SmFBX202, (Figure 5B). These results suggest that the F-box candidate proteins interact with specific Skp1 proteins.

### 2.10. SmFBX131 and SmFBX230 Could Improve the Resistance to Bacterial Wilt

**Figure 6:**
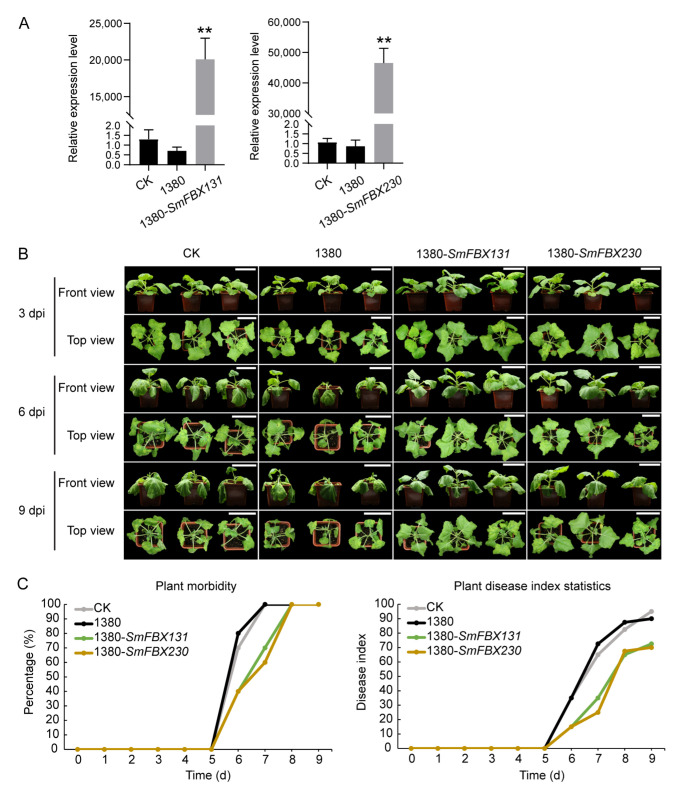
Transient overexpression of *SmFBX131* and *SmFBX230* could improve plant resistance to bacterial wilt. (**A**) The expression levels of *SmFBX131* and *SmFBX230* in plants. CK (control), tobacco leaves that were injected with water. 1380, tobacco plants whose leaves were injected with Agrobacterium harboring an empty vector (pCAMBIA-1380). 1380-*SmFBX131* and 1380-*SmFBX230*, the *SmFBX131*-transient overexpression and *SmFBX230*-transient overexpression plants, respectively. Data are expressed as the mean ± SEM values of three biological replicates. The method of calculation was 2^−∆∆ct^. ** *p* < 0.01. Student’s *t*-test. (**B**) The plant phenotype after 3, 6, and 9 days of inoculation with *Ralstonia solanacearum.* (**C**) The plant morbidity and disease index statistics of plants after inoculation with *R. solanacearum* are summarized over 9 days. At least 10 biological replicates were performed. Dpi, days after inoculation; SEM, standard error of the mean. Scale bars indicate 10 cm.

To determine whether *SmFBX131* and *SmFBX230* are associated with BW resistance in eggplant, *SmFBX131* and *SmFBX230* were transiently overexpressed in tobacco, followed by inoculation of the putative transgenics with *R. solanacearum*. Results showed that the wilting symptoms of the control plants (control check (CK) and empty vector (1380)) plants were more evident after *R. solanacearum* inoculation than wilting symptoms in the *SmFBX131*-transient and *SmFBX230*-transient overexpression plants (Figure 6A,B). When the control plants were all diseased, the plant morbidity of *SmFBX131*- and *SmFBX230*-transient overexpression plants was 70% and 60%, respectively (Figure 6C). On the 9th day after *R. solanacearum* inoculation, the disease index of control groups (CK and 1380) was 95 and 90, respectively. In contrast, the disease index of *SmFBX131*- and SmFBX230-transient overexpression plants were 72.5 and 70, respectively (Figure 6C, Supplementary Table S15). These results suggest that *SmFBX131* and *SmFBX230* could improve the resistance of eggplant to BW.

## 3. Discussion

The F-box gene superfamily plays a critical role in plant growth and stress resistance [40,41]. Genome-wide analyses of the F-box gene superfamily have been performed in more than 20 species [9,10,12,42]. However, the F-box gene superfamily has rarely been analyzed in eggplant. Eggplant, an important horticultural crop with economic benefits, is a significant resource of stress-resistance genes. Many eggplant varieties resist biotic or abiotic stresses [43]. This study identified and analyzed 389 candidate members of the F-box gene superfamily from the eggplant genome.

Many plants have F-box proteins with the same C-terminal domain types. It is worth noting that different C-terminal domains represent different functions. For example, the FBA domain is related to ABA [44], the Kelch domain can regulate plant metabolism and tolerance [45,46], and the PP2 domain is primarily associated with plant immunity [47,48] and responses to abiotic stress [49]. In this study, we identified 16 types of C-terminal domains after comparing the F-box candidate proteins from *A. thaliana,* rice, tomato, and eggplant. LRR, LRR_FBD, Kelch, FBA, TUB, and FBD accounted for a large proportion, but there were differences in the numbers between the four species. Arm, Cupin_8, selI, and WD40 were found in a few domains of F-box candidate proteins, and these findings were stable among the four species (Figure 1). In addition, there were more F-box candidate proteins with Kelch and LRR domains in eggplant than in tomato, and more DUF295, FBA, and FBD domain types of F-box candidate proteins in tomato than in eggplant. The difference in the number and type of C-terminal domains could be one of the reasons why eggplant has more tolerant varieties than tomato. Most of the F-box proteins in various plants, such as rice [9], chickpea [13], soybean [10], and pear [15], have an unknown domain at the C-terminus. Therefore, we hypothesized that many retained C-terminal domains might have fundamental roles in plant growth and development.

Some domains were substantially duplicated in a specific species, such as LRR_FBD and FBA in *A. thaliana* and DUF295 in rice (Figure 1, Supplementary Table S2). The F-box superfamily has undergone extensive gene duplication during evolution, particularly in the production of tandem repeats, which have been reported during the evolution of F-box genes in many species [11,18]. Gene duplication leads to an increase in the same types of domains in F-box genes. The present study found that the repeated genes had different expression patterns in eggplant (Supplementary Table S3), which suggests that they have different roles. The domain of the F-box protein could be spliced by a conserved N-terminal F-box domain and one or several rapidly evolving domains at the C-terminus. Results showed the presence of multiple domains at the C-terminus, such as Kelch and PAS_9, zf_MYND and sel1, Transp_inhibit and LRR, and FBD and LRR, with these types of domains also appearing solely in the F-box genes (Supplementary Table S2). Therefore, the existence of the unknown domain F-box at the C-terminus could be the transitional state of the F-box superfamily during evolution. Similar inferences about “domain combinations” have also been reported by Bashton and Chothia [50] and Vogel et al. [51].

The F-box is a substrate receptor of the multi-subunit E3s CUL^SCF^. A previous study reported that the Skp1 subunit is the critical subunit that connects the F-box and CUL1 [52]. This study found that the candidate F-box proteins could interact with the representative SKP1-like proteins. One SmFBX candidate protein can specifically bind to different Skp1-like proteins [53]. Currently, the numbers of Skp1-like genes are also rapidly changing. For example, there are 19 ASK (*A. thaliana* SKP1-like) genes in *A. thaliana*, 28 OSK (*Oryza* SKP1-like) genes in rice [54], and 19 SSK (*Solanum* SKP1-like) genes in tomato [55]. Here, we identified 13 Skp1-like genes in eggplant. We hypothesized that the change in Skp1 was accompanied by a change in the F-box genes. Different F-box proteins and Skp1 could form a potential SCF combination that targets more substrate proteins.

Tissue-specific expression analyses are employed to explore the functions of genes in plant growth and development. In this study, the F-box candidate genes were stratified into 10 clusters (Figure 2, Supplementary Tables S5–S7). Notably, homologous F-box genes could have similar functions. For example, *SmFBX241* and *SmFBX353* in cluster 8 are homologous to *MAX2* and *ORE9*, respectively. *MAX2* was found to have an essential role in the light signaling pathway in *A. thaliana* [56] and apple [25], whereas *ORE9* has been shown to regulate plant leaf senescence [57]. *SmFBX19* is homologous to *FKF1* in cluster 5. In *A. thaliana*, *FKF1* was found to modulate the plant flowering time by regulating the abundance of COP1 and DELLA proteins [58,59]. In addition, the F-box candidate genes in cluster 9 were associated with flower development, and *SmFBX172* in cluster 9 is homologous to the plum F-box gene *PslSLY1*. *Ps1SLY1* regulates plant germination, stem elongation, flower structure, and fruit development through gibberellin (GA) signaling [60].

We also found that *SmFBX11* (domain types: LRR), *SmFBX13* (N/A), *SmFBX49* (GRAS), *SmFBX172* (N/A), and *SmFBX193* (PP2) positively responded to heat stress in eggplant. *SmFBX75* (N/A), *SmFBX111* (N/A), and *SmFBX289* (FBA) negatively responded to heat stress (Figure 3). Previous studies have shown that *SmFBX11*, *SmFBX172*, and *SmFBX289* encode GID2, EBF1, and F-box/Kelch repeats, respectively. GID2 regulates the GA pathway [61], EBF1 is associated with ethylene (Eth) [62], and the F-box/Kelch repeats protein modulates the content of ABA [63]. *TaFBA1,* a homolog of *SmFBX372* in cluster 6, could positively regulate heat resistance in wheat [23]. Our RNA-seq data showed that HTT induced the expression of SmFBX372 at 12 h (Figure 4A, Supplementary Table S8).

The F-box genes have been extensively studied in heat resistance, salt tolerance, and drought resistance, but their role in resistance to BW has rarely been explored. Herein, we identified *SmFBX23* (domain types: Kelch), *SmFBX131* (Kelch), *SmFBX219* (N/A), and *SmFBX230* (N/A) as candidate genes that positively respond to BW (Figure 4). We also evaluated the function of *SmFBX131* and *SmFBX230*. Results revealed that transient overexpression of *SmFBX131* and *SmFBX230* in tobacco could improve the resistance to BW, suggesting that *SmFBX131* and *SmFBX230* positively regulate the plant resistance to *R. solanacearum* (Figure 6). In contrast, *SmFBX48* (LysM) and *SmFBX275* (LRR) negatively responded to eggplant BW (Figure 4). It should be noted that SmFBX23 and SmFBX131 are F-box/Kelch repeat-type proteins. Previous studies have shown that the F-box/Kelch repeat proteins can positively respond to the resistance of *Vitis pseudoreticulata,* the wild Chinese grape species, against powdery mildew [64]. It has also been reported that SmFBX219 and SmFBX275 are F-box/LRR repeat proteins that can function in disease resistance [65,66]. In addition, *MAX2*, the homologous gene of *SmFBX241*, positively regulates bacterial resistance in *A. thaliana* [26]. This is consistent with our findings that *R. solanacearum* could induce the expression of some genes in cluster 3.

## 4. Material and Methods

### 4.1. Gene Identification and Screening

The eggplant genome V4 was downloaded from the Solanaceae Genome website (https://solgenomics.net/, accessed on 16 August 2022) [38]. In addition, the *A. thaliana*, tomato, and rice genomes were downloaded from the National Center for Biotechnology Information (NCBI, https://www.ncbi.nlm.nih.gov/, accessed on 16 August 2022), with accession numbers GCF_000001735.4, GCF_000188115.4, and GCF_001433935.1, respectively. For each species, we only retained the protein sequences that correspond to the longest alternative splicing isoform. Next, we searched for a sequence that matched the PF00646.34 model (https://www.ebi.ac.uk/interpro/download/Pfam/, accessed on 20 August 2022) in the protein sequence using the HMMER 3.2.1 [67] software, with “-E = 1 × 10^−2^” as the search parameter. To obtain a more comprehensive F-box candidate protein set, PSI-BLAST was used to scan all the proteins based on the F-box proteins obtained in the previous step. The obtained F-box protein sequences were then used for subsequent analyses. Specifically, the sequence of the F-box domain was extracted from the F-box protein obtained in the first step, followed by removing the redundancy using CD-HIT (-c = 0.8). Finally, each F-box domain sequence was aligned to all the proteins of the four species using PSI-BLAST (-evalue = 1 × 10^−2^, -inclusion_ethresh = 1 × 10^−2^, -num_iterations = 10).

### 4.2. Analyses of Domains and Conserved F-Box Domains

The F-box domain sequences of each F-box gene from each species were separately aligned using T-Coffee [68], followed by visualization of their conserved sites using Web-Logo. The C-terminal domains were identified by Pfamscan.pl v. 1.6 (default parameters) using the Pfam-A database (https://www.ebi.ac.uk/interpro/download/Pfam/, accessed on 1 September 2022) [69]. The F-box genes were then classified based on their C-terminal domains.

### 4.3. Chromosome Distribution

TBtools [70] was used to map the distribution of F-box genes on the chromosomes.

### 4.4. Duplicate Gene Statistics and Intra-Species and Inter-Species Collinearity Analyses

The intra-species (eggplant compared with eggplant) collinearity analysis was performed based on the duplication pattern of the F-box candidate genes. We clustered the F-box candidate genes by CD-HIT in a sequence similarity of 90%. F-box candidate genes in the same cluster were considered duplicated and were visualized using Circos. For inter-species (eggplant compared with the other three species) collinearity analysis, JCVI toolkits [71] were used to perform the orthologous gene matching and visualization. In the “jcvi.compara.catalog” step, the “cscore” parameter was set to 0.99.

### 4.5. Gene Expression Analysis

Data for tissue-specific expression were obtained from previous eggplant genome research [72]. The raw transcriptome data was downloaded and re-analyzed to obtain an expression matrix. The detailed analysis pipeline was as follows: the reads data were first aligned to the eggplant genome using STAR 2.7.9a software [73], quantified using featureCounts [74], and then normalized using DESeq2 [75]. The RNA-seq data for the abiotic treatment was obtained from our laboratory (unpublished results), whereas the RNA-seq data for the biotic treatment were obtained from a previous study [76]. We used the pre-processed expression matrices in these two datasets where the gene IDs differed from those of the eggplant genome V4. To ensure that the gene IDs of each expression matrix were consistent, we performed ID mapping with an RBH tool integrated into MMSeqs2 [77]. The “hclust” function in R 4.0.3 software was used to perform the gene cluster analysis in which the clustering method was set to “complete,” and the distance matrix was computed using the “Euclidean” method. Finally, the Circos heatmap was plotted using the “ComplexHeatmap” package in R software [78].

### 4.6. Experimental Materials

The eggplant BW-resistant (E31) and BW-sensitive (E32) inbred lines, eggplant heat-resistant (390) and heat-sensitive (Hua Mo (HM)) inbred lines, and *N. benthamiana* seedlings used in this study were provided by the School of Horticulture, South China Agricultural University (Guangzhou, China). The eggplant seedlings were grown to 3–4 true leaves under 25 °C, 16 h of light, and 22 °C, 8 h of darkness. The tobacco seedlings were grown to 6–7 leaves under 22 °C, 14 h of light, and 20 °C, 10 h of darkness. The *R. solanacearum* (strain GMI1000) was provided by South China Agricultural University. The yeast two-hybrid (Y2H) competent cells were Y2H Gold.

### 4.7. R. solanacearum Inoculation

*R. solanacearum* was streaked on 2,3,5-triphenyltetrazolium chloride (TTC) solid media [79] and incubated at 28 °C for two days. Next, a single colony was picked, inoculated on TTC medium, and incubated at 28 °C on a rotary shaker at 200 rpm until the OD_600_ reached 0.6. A third of the eggplant roots were removed using a pair of scissors, followed by watering the plants with 50 mL of the bacterial solution. Notably, the control eggplant seedlings were watered with the same amount of water.

### 4.8. High-Temperature Treatment of Eggplant

For the high-temperature treatment experiment, eggplant seedlings were subjected to 43 °C during the day and 38 °C at night, with a 16 h:8 h light:dark photoperiod. Total RNA was extracted from eggplant leaves after 0, 1, 6, and 12 h. The experiment had at least three biological replicates.

### 4.9. RNA Extraction and Data Processing

Total RNA was isolated as described by Qiu et al. [80]. cDNA was synthesized using EZ-press Cell to cDNA Kit PLUS (B0003) (EZBioscience, Roseville, MN, USA), and the qRT-PCR experiments were performed as described by Qiu et al. [80]. Moreover, relative mRNA expression was normalized to Cyclophilin (eggplant) or NtActin (*Nicotiana benthamiana*) and calculated using the 2^−∆∆ct^ method as previously described [81]. The primers used are listed in Supplementary Table S17.

### 4.10. Phylogenetic Analysis of the F-Box Candidate Gene Families

We only used the F-box domain part of each F-box amino acid sequence to construct the phylogenetic tree. Precisely, the sequences were aligned by T-Coffee v13.45.60.cd84d2a (-mode psicoffee), followed by tree inference using IQ-TREE v1.6.12 (-m TEST –bb 1000) [82]. The phylogenetic trees were visualized by iTOL [83].

### 4.11. Yeast Two-Hybrid Assay

The *A. thaliana* Skp1 protein was used as the seed sequence to search the eggplant proteome. The search identified 13 Skp1-like proteins, among which five proteins were screened and cloned into the pGBKT7 vector. We also selected seven representative eggplant F-box genes based on the phylogenetic tree and cloned them into the pGADT7 vector. The yeast two-hybrid assay was then performed according to the manufacturer’s instructions (Cat. No. 630489; Clontech, Mountain View, CA, USA). The specific primers used are shown in Supplementary Table S17.

### 4.12. Transient Overexpression Assay

The full-length coding sequences of *SmFBX131* and *SmFBX230* were cloned into the *Xba* I and *BamH* I sites of the pCAMBIA-1380 vector. The recombinant vector and pCAMBIA-1380 vector were transformed into *A. tumefaciens* strain GV3101, which was then cultured in YEP medium (10 g/L tryptone, 10 g/L yeast extract, 5 g/L NaCl) until the OD_600_ reached 0.6. After centrifugation at 5000 rpm for 5 min, bacterial cells were collected and resuspended in the infection solution (10 mM MgCl_2_, 10 mM MES (pH = 5.6), 100 μM AS). Next, the infection solution was infiltrated into the leaves of six- or seven-leaf-old *N. benthamiana* seedlings using a 1 mL needleless syringe until the leaves of the seedings became water-soaked. The control group was injected with water (CK), and the infection solution contained an empty vector (1380). After injection, the seedlings were incubated in the dark (22 °C, 14 h; 20 °C, 10 h) for one day and then grown normally for 3–4 days (22 °C, 14 h light; 20 °C, 10 h dark). Each treatment had at least 10 biological replicates. The primers used are shown in Supplementary Tables S16 and S17.

### 4.13. Calculation of Morbidity and Disease Index of Tobacco Bacterial Wilt

The tobacco BW incidence grade was determined as described by Scherf et al. [84], where 0, healthy; 1, >1/4 leaves wilted; 2, 1/4–1/2 leaves wilted; 3, 1/2–3/4 leaves wilted; 4, >3/4 leaves wilted or plant death.

Tobacco morbidity = diseased plants/total plants.

Disease index = ∑ (incidence grade× the number of diseased plants of the corresponding grade)/(highest disease grade× total plants) ×100.

## 5. Conclusions

Overall, this study identified 389 eggplant F-box candidate genes through a PSI-BLAST-based method. The motifs, gene structures, and chromosomal locations of candidate *SmFBX* were characterized. The conserved sites of the F-box domain and the C-terminal domain type analyses of the F-box candidate proteins showed that the N-terminal F-box domain is conserved, whereas the C-terminal domain is diverse. A collinearity analysis within the eggplant genome showed that most of the F-box candidate repeat genes in eggplant were composed of tandem repeats. The collinearity analyses between the four species provide insights into the evolutionary signature of F-box candidate genes. The *SmFBX* candidate genes that may be associated with heat tolerance and BW resistance were screened by analyzing the expression of *SmFBX* candidate genes under heat and disease stresses. Furthermore, some representative F-box candidate proteins were found to bind the Skp1-like proteins. In addition, transient overexpression of *SmFBX131* and *SmFBX230* in tobacco demonstrated that the genes positively regulate plant BW resistance. This study lays the foundation for the functional analysis of eggplant F-box genes and provides valuable insights for breeding superior eggplant varieties.

## Data Availability

Transcriptomes obtained after high-temperature treatment of eggplant seedlings and transcriptome data sets obtained after *Ralstonia solanacearum* treatment analyzed during the current study are available from the corresponding authors on reasonable request. Additional data generated or analyzed during this study are included in this article [and its Supplementary Information files].

## Supplementary Materials

The following supporting information can be downloaded at: https://www.mdpi.com/article/10.3390/ijms232416049/s1.
