# Supplementary material for: Genome-Wide Analysis and Characterization of Eggplant F-Box Gene Superfamily: Gene Evolution and Expression Analysis under Stress
Source: Int J Mol Sci. 2022 Dec 16;23(24):16049. doi: 10.3390/ijms232416049 (PMC9780924; doi:10.3390/ijms232416049)
Supplement: Supplementary file 1 [file ijms-23-16049-s001.zip › Supplement Figures .pdf]

Supplementary Figures

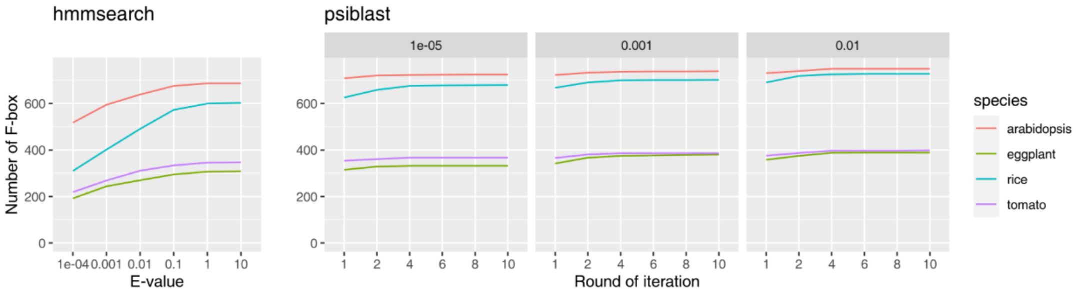

Supplementary Figure S1. The number of F-box genes in *Arabidopsis*, rice, tomato, and eggplant converged in rescreening. The left figure shows the number of F-box genes at different thresholds screened by HMM method, and the three figures on the right show the number of F-box genes filtered by PSI-BLAST at HMM thresholds of 1e-5, 0.001, and 0.01. The abscissa represents the number of F-box genes screened for each species by the PSI-BLAST method, and the ordinate represents the number of F-box genes.

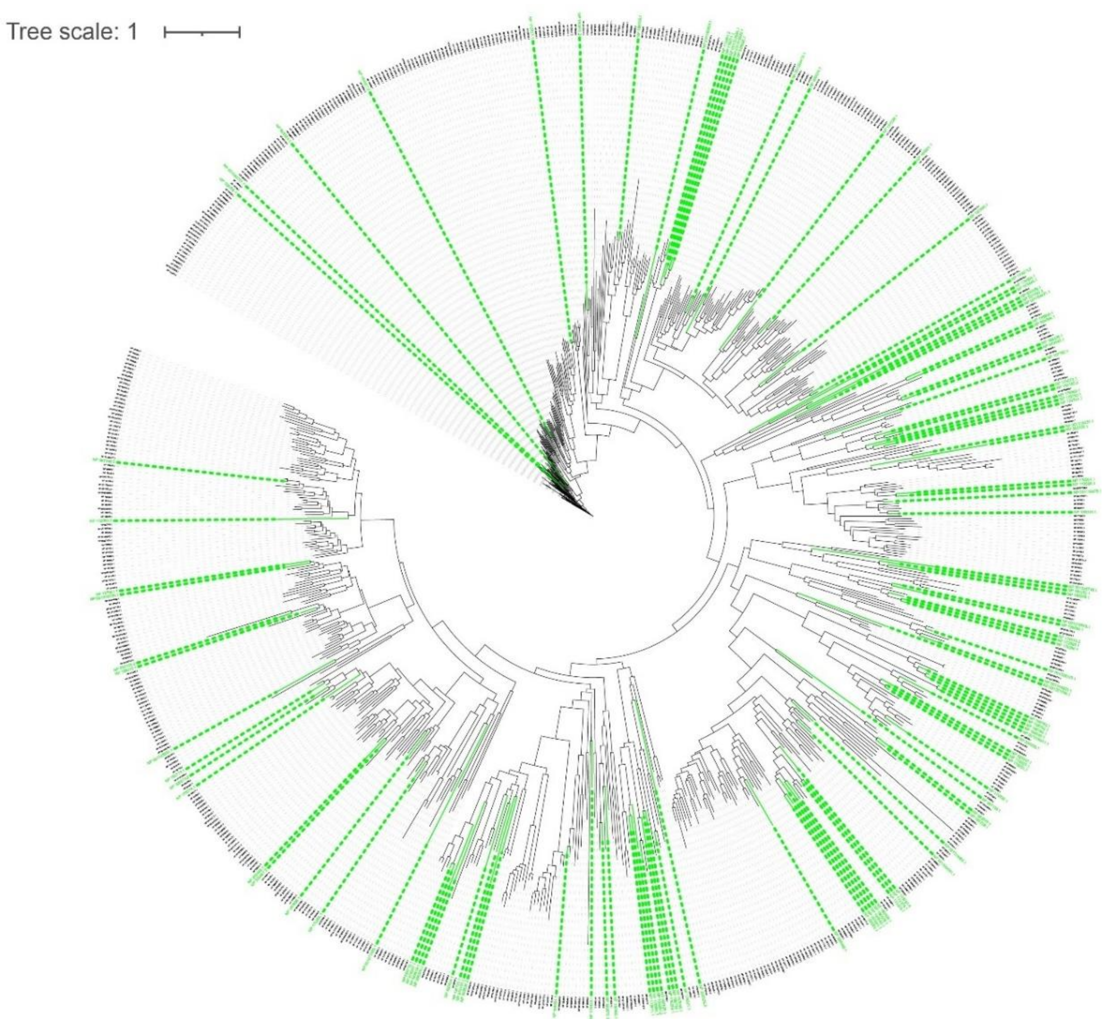

Supplementary Figure S2. The phylogenetic tree of *Arabidopsis* F-box proteins identified by PSI-BLAST method. F-box proteins not identified by the HMM method were marked in green.

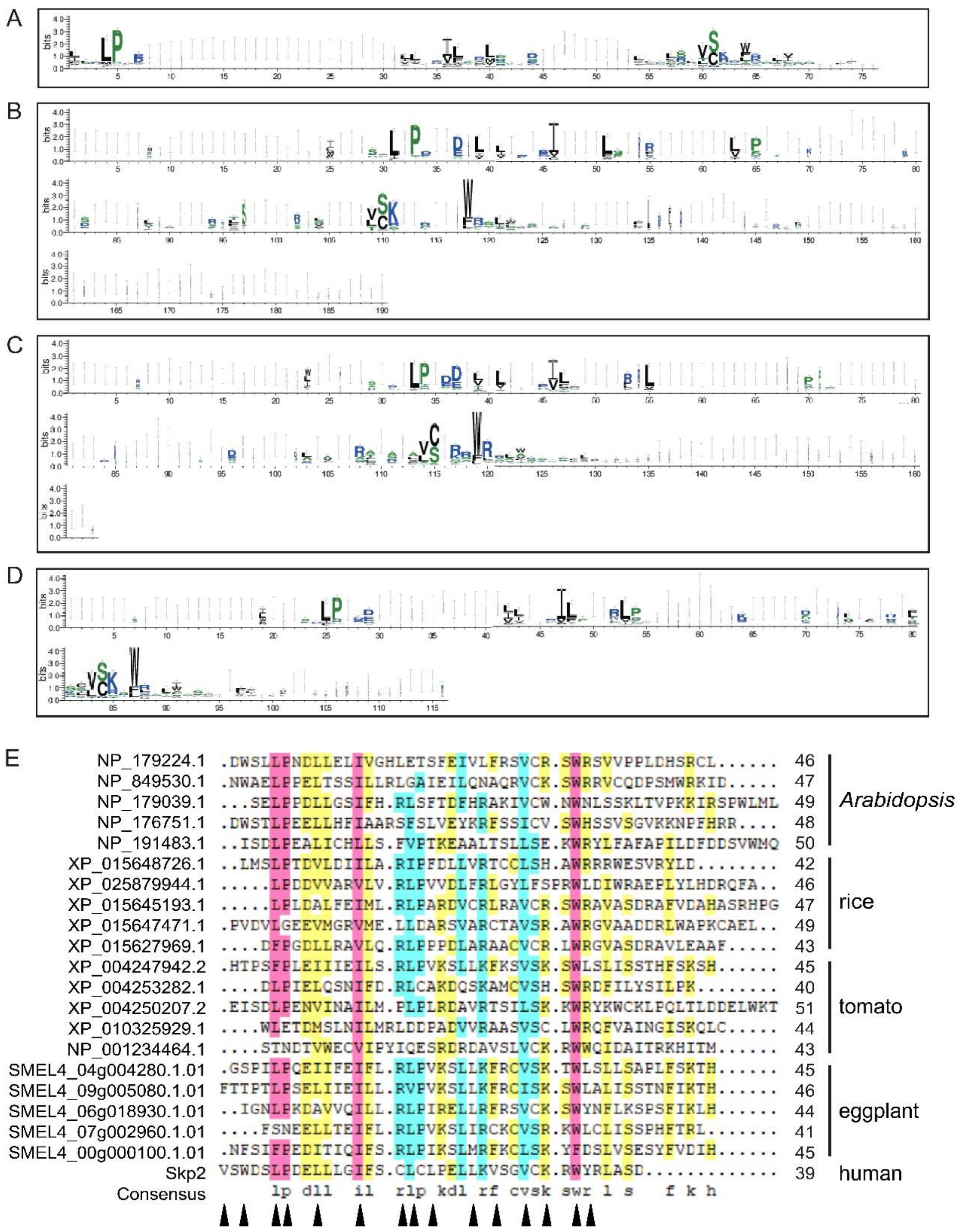

Supplementary Figure S3. F-box sequences are conserved. (A)-(D) Conserved sequence analysis of F-box in *Arabidopsis*, rice, tomato, and eggplant, respectively, the larger the letter, the more conserved the corresponding amino acid at the position. (E) Comparison of F-box conserved sequence genes in *Arabidopsis*, rice, tomato, and eggplant with the human F-box protein Skp2. Arrows mark amino acid positions necessary for the Skp/F-box interaction between human Skp1 and Skp2 (Gagne et al., 2002). Conserved or similar amino acids are marked with different colored boxes.

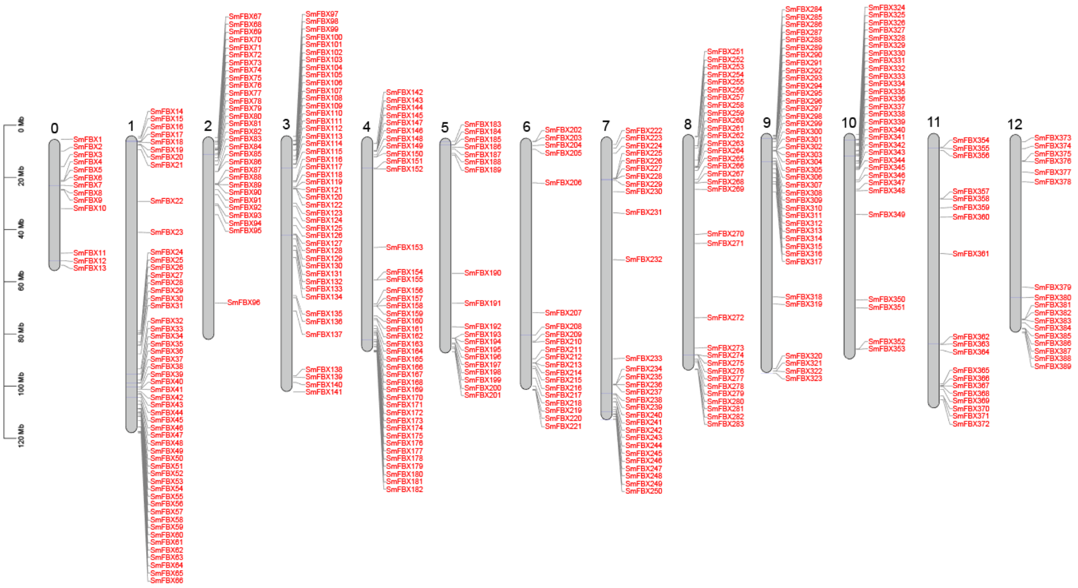

Supplementary Figure S4. Schematic diagram of the location of the F-box genes identified in eggplant on the chromosome. The grey cylinders represent the chromosomes, and the numbers above represent the chromosome numbers.

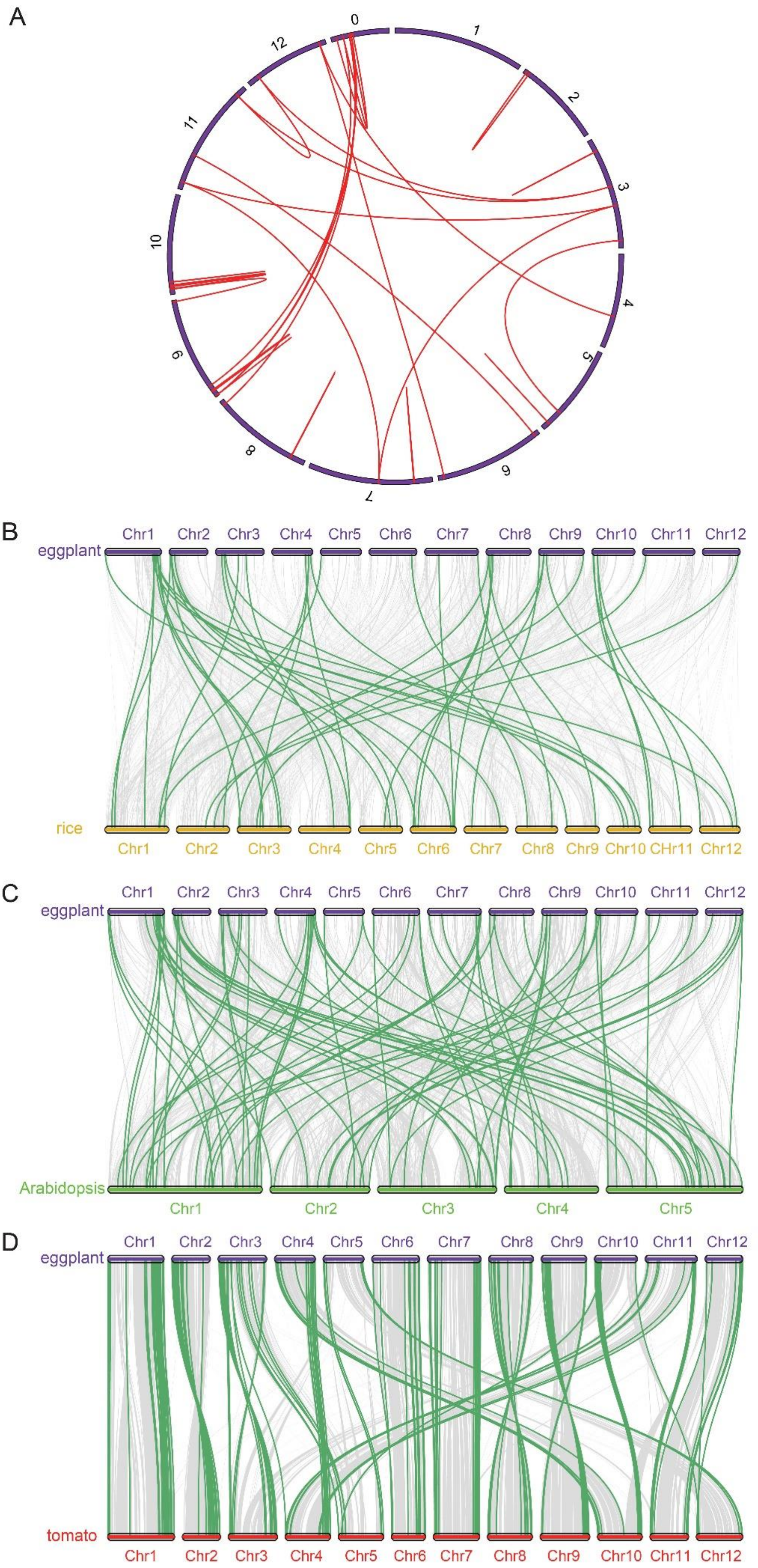
A
0
1
2
3
4
5
6
7
8
9
10
11
12
B
eggplant
Chr1 Chr2 Chr3 Chr4 Chr5 Chr6 Chr7 Chr8 Chr9 Chr10 Chr11 Chr12
rice
Chr1 Chr2 Chr3 Chr4 Chr5 Chr6 Chr7 Chr8 Chr9 Chr10 CHr11 Chr12
C
eggplant
Chr1 Chr2 Chr3 Chr4 Chr5 Chr6 Chr7 Chr8 Chr9 Chr10 Chr11 Chr12
Arabidopsis
Chr1 Chr2 Chr3 Chr4 Chr5
D
eggplant
Chr1 Chr2 Chr3 Chr4 Chr5 Chr6 Chr7 Chr8 Chr9 Chr10 Chr11 Chr12
tomato
Chr1 Chr2 Chr3 Chr4 Chr5 Chr6 Chr7 Chr8 Chr9 Chr10 Chr11 Chr12

Supplementary Figure S5. Collinearity analysis of homologous genes in the F-box family within eggplant, and between eggplant and *Arabidopsis thaliana*, rice, and tomato. (A) Collinearity analysis of homologous genes in the F-box family within eggplant. Homologous genes in the F-box family are represented by red lines, purple figures represent chromosomes, and the numbers outside correspond to chromosome numbers. Collinear analysis of F-box genes of eggplant and rice (B), *A. thaliana* (C) and tomato (D), respectively. Purple, yellow, green, and red cuboids represent the chromosomes of eggplant, rice, *A. thaliana*, and tomato, respectively. The numbers represent chromosome numbers. The grey lines refer to all the homologous genes between the two species. The green line represents all the predicted homologous genes that belong to the F-box family between the two species.

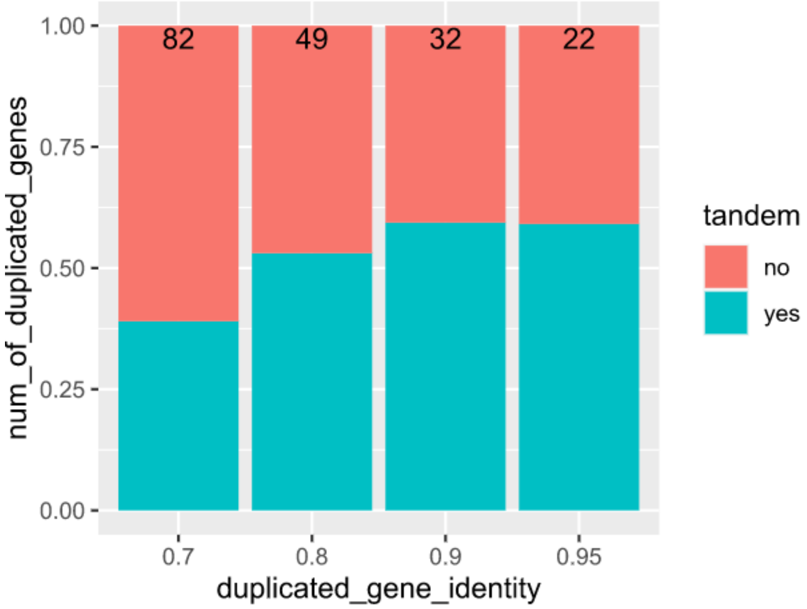

Supplementary Figure S6. The proportion of tandem repeats in repeat patterns at different screening thresholds. The abscissa is the identification threshold of duplicated genes, and the ordinate is the proportion of tandem duplication and non-tandem duplication models. Green-blue bars represent tandem repeat events, and red represents non-tandem repeat events. The numbers above the graph represent the total number of duplicate genes that meet this threshold.

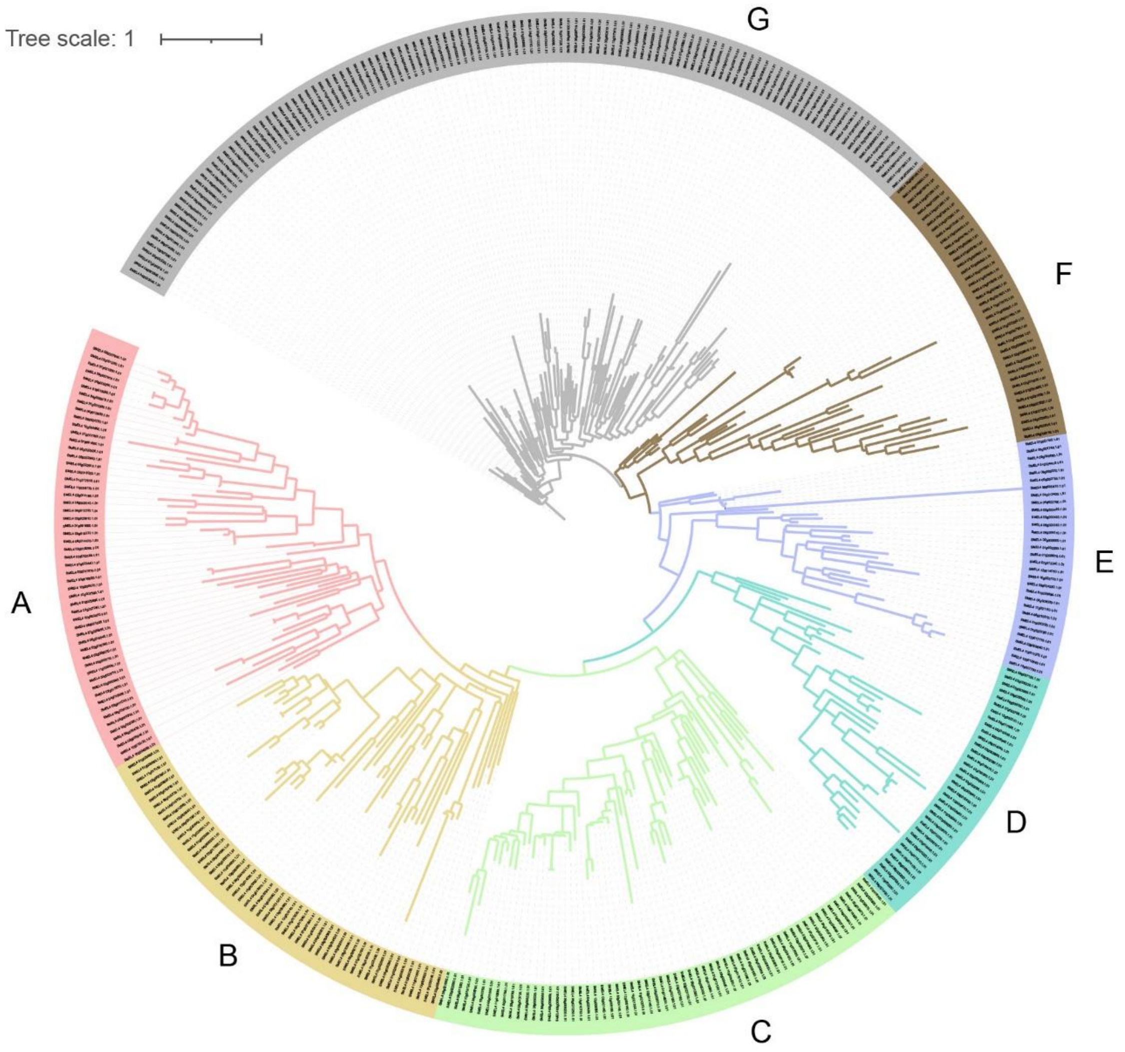

Supplementary Figure S7. The phylogenetic tree of *SmFBX* candidate genes was constructed. According to phylogenetic analysis, *SmFBX* candidate genes are divided into seven different subfamilies, which are represented by A-G. Each subfamily is represented by a different color block.
